# The Tidal Wave of Sanitation

**Published:** 1908-07

**Authors:** 


### EDITORIAL DEPARTMENT.

### The Tidal Wave of Sanitation.

The great movement for sanitary reform is rolling like a tidal wave over the whole country. It originated with the American Public Health Association; was taken up and given impetus by the American Association for the Advancement of Science, and at a memorable meeting at Ithaca, New York, June 30, 1906, was fairly launched and given a boom by the most remarkable and valuable paper yet written. It was by Professor J. P. Norton, of Yale, Assistant Professor of Economics, the title being "The Economic Advisability of Inaugurating a National Organization of Health." A copy of this great paper can be had for the asking by writing to Professor Norton, New Haven, Conn.

Immediately there was inaugurated the American Health League, which, in collaboration with the Committee of One Hundred appointed by the Association for the Advancement of Science, is pushing the good work. The movement has at last arrested public attention—even that of Congress (see Department of Abstracts), and sanitary science may now be said to be well to the fore.

The public are beginning to understand the difference between sanitary science and medical science. The one aims at the prevention of disease and injury by removing the cause; the other at curing the results of causes that should have been removed; they begin to realize that the first is easier and cheaper than the second, and that in the first the expense falls on the government, and the individual or community is spared the cost of sickness, and, often, death. The States are pushing the campaign and Texas aims to be well in the lead.

The first step in the desired reform is registration; a perfected system of vital statistics. It is "the mother of sanitary science." The Twenty-eight Legislature passed the bill creating a bureau of vital statistics (drafted by the writer of this, as Chairman of the State Medical Association's Committee on Legislation), but it was emasculated by an unthinking Senator who caused to be cut out the small appropriation it carried. This bill will be perfected by the next Legislature, and Texas will then be taken into the "registration area" of the United States. She will then do her share in the life-saving work to which she has set herself, by other methods than medical practice. It is expected that six hundred Texas doctors will attend the great tuberculosis Congress at Washington in September as delegates; recruits in the war against the "great white waste."

In this connection the following data from the recently issued mortality statistics for 1906 (U. S. census) will be found interesting, especially as showing what has been accomplished since last report, in gathering reliable statistics. For instance: The census reports for 1900 showed a population (estimated) of 75,994,575. Of this population only from 37 to 40 per cent was represented by vital statistics from ten States then constituting the registration area. Since that report five great States have perfected their system and have been added to the registration area, and thus have been added reliable data as to births and deaths in 48.8 per cent (nearly one-half) of the entire population, although, even with this addition of five States, the registration area embraces only fifteen of the forty-six States.

The States which have been added are California, Colorado, South Dakota, Maryland and Pennsylvania. Texas must be represented in next report.

The election of Colonel Gorgas to the Presidency of the American Medical Association, the foremost sanitarian of the world and America's greatest benefactor, gives emphasis to the awakening of the American people to the necessity of substituting prevention for (efforts at) *cure* in dealing with disease.

